# Association of Levels of Mannose-Binding Lectin and the *MBL2* Gene with Type 2 Diabetes and Diabetic Nephropathy

**DOI:** 10.1371/journal.pone.0083059

**Published:** 2013-12-20

**Authors:** Nana Zhang, Maoqiang Zhuang, Aixia Ma, Guochang Wang, Ping Cheng, Yajun Yang, Xiaofeng Wang, Juan Zhang, Xingdong Chen, Ming Lu

**Affiliations:** 1 Clinical Epidemiology Unit, Qilu Hospital of Shandong University, Jinan, Shandong, P. R. China; 2 MOE Key Laboratory of Contemporary Anthropology, School of Life Sciences and Institutes of Biomedical Sciences, Fudan University, Shanghai, P. R. China; 3 CMC Institute of Health Sciences, Taizhou, Jiangsu, P. R. China; Boston Children’s Hospital and Harvard Medical School, United States of America

## Abstract

**Objective:**

To investigate the association of Mannose-binding lectin (*MBL*) and the *MBL2* gene with type 2 diabetes and diabetic nephropathy and the influence of *MBL2* polymorphisms on serum *MBL* levels.

**Methods:**

The study population included 675 type 2 diabetic patients with or without nephropathy and 855 normoglycemic controls. The single nucleotide polymorphisms (SNPs) of rs1800450, rs1800451, and rs11003125 of the *MBL2* gene were determined by the Multiplex Snapshot method. Serum *MBL* levels were measured by enzyme-linked immune sorbent assay.

**Results:**

Rs1800450 and rs11003125 SNPs demonstrated strong linkage disequilibrium in the study population (r^2^ = 0.97). The haplotypes constructed from the G allele of rs1800450 and the C allele of rs11003125 increased the risk for type 2 diabetes (*OR* = 1.2, 95% *CI* = 1.1–1.4, *P = *0.01). For rs1800450, GG and GA genotypes were associated with type 2 diabetes (*P = *0.02, 0.01, respectively). For rs11003125, the GC genotype frequency was significantly different between patients and controls (18.1% vs. 24.9%, *P = *0.001). Analyses of genotypes and allele frequency distributions among patients with normal UAE, microalbuminuria, and macroalbuminuria showed that there was no obvious evidence of association between the *MBL2* gene and diabetic nephropathy. Subjects with the GG genotype of rs1800450 and the CC genotype of rs11003125 had much higher serum *MBL* levels.

**Conclusions:**

The rs1800450 and rs11003125 SNPs of the *MBL2* gene have strong linkage disequilibrium and are associated with type 2 diabetes in the North Chinese Han population. No association was observed between the *MBL2* gene and diabetic nephropathy. Subjects with the GG genotype of rs1800450 and the CC genotype of rs11003125 had much higher serum MBL levels. An association between elevated serum *MBL* and diabetic nephropathy was also observed.

## Introduction

Epidemiological statistics showed that the age-standardized prevalence of diabetes (which included both previously diagnosed and undiagnosed diabetes) and prediabetes were 9.7% and 15.5% among Chinese adults, respectively, in 2008 [1]. The main hazard of diabetes comes from its complications. Among the complications, diabetic nephropathy is a common and devastating microvascular complication and its pathogenesis is still poorly understood [2], although complement activation and inflammation have been suggested to play essential roles in the pathogenesis of diabetes and diabetic vascular lesions [3]–[5].

Mannose-binding lectin (*MBL*, also known as mannan-binding lectin) is a C-type lectin secreted by the liver and is a component of the innate immune defense system [4]. *MBL* deficiency is associated with autoimmune, inflammatory, infectious, and vascular disease. Upon binding to specific carbohydrate structures, *MBL* activates the third pathway of complement (the lectin pathway) and aggravates inflammation [6]. Serum *MBL* levels are significantly elevated in patients with type 1 diabetes [3], [4], and even higher in those patients with microvascular and macrovascular complications [7], [8]. The median serum *MBL* level in healthy Caucasians is 800–1000 ng/ml [9], [10], but *MBL* levels vary widely from person to person mainly because of frequently occurring polymorphisms within exon1, as well as in the promoter region of the *MBL2* gene on chromosome 10. Three non-synonymous SNPs in exon1 named alleles B(G54D/rs1800450), C(G57E/rs1800451), and D(R52C/rs5030737) considerably decrease serum *MBL* levels due to incorrect assembly of the mature *MBL* protein [11].

The first aim of the present study was to investigate the association of *MBL2* gene polymorphisms with type 2 diabetes and diabetic nephropathy in a Chinese Han population. The secondary aim was to validate the association between diabetic nephropathy and elevated *MBL* levels as previously studies have suggested. Third, the association between *MBL2* polymorphisms and serum *MBL* levels was considered.

## Subjects and Methods

### Ethics Statement

This study was approved by the Ethics Committee of Qilu Hospital, Shandong University. All participants gave their written informed consent prior to participation.

### Subjects

1530 subjects (675 type 2 diabetic patients and 855 controls) were recruited from Qilu Hospital of Shandong University, from March 2010 to October 2011. All study subjects were asked to complete an epidemiological survey by face to face interview (including their basic information, clinic indexes, and potential risk factors of diabetes). Subjects who reported a history of autoimmune diseases, inflammatory diseases, infectious diseases, type 1 diabetes or gestational diabetes were excluded. For normoglycemic controls, 855 subjects, age- and sex-matched with patients, were randomly collected from the Physical Examination Center of Qilu Hospital. Twenty-four hour urinary albumin excretion (UAE) is considered as the gold standard method to assess albuminuria levels [12]. The nephropathy status of patients was classified into three groups based on the UAE. Microalbuminuria was defined as UAE between 20 and 200 µg/min or between 30 and 300 mg/24 h, macroalbuminuria was defined as above 200 µg/min or 300 mg/24 h in at least two of three consecutive 24-h urine collections [8], [13], [14]. Current smokers were defined as those who reported smoking at the time of interview and had a smoking history for more than 1 year with at least one cigarette per day. Subjects who did not meet the criteria of current smokers were defined as non-smokers. Alcohol drinkers were defined as those who reported drinking at the time of interview and had a drinking history for more than 1 year, drinking, at least three times (>50 ml alcohol per time) per week. Non-drinkers were defined as those who had never drunk or did not meet the criteria of drinkers.

### SNP Selection and Genotyping

Six SNPs in the *MBL2* gene are known to be associated with variation in quantity and/or function of serum *MBL*. Three base substitutions in exon 1 in codons 54 (B)/rs1800450, 57 (C)/rs1800451 and 52 (D)/rs5030737 decrease the level and function of *MBL* [15]. The mutation of rs1800450 is frequent in healthy Caucasians, Asians and Inuit from East Greenland, whereas it is rare in East Africa [15]. Some studies suggested that rs1800450 is the main variation in different Chinese nationalities, and rs1800451 and rs5030737 are not found in the same population [16], [17]. The mutation of rs1800451 is frequent in Africa, and it has a very low frequency in Caucasians, Asians and pure Inuit. We chose rs1800451 to verify the consistency with previous studies. The mutation of rs5030737 has a very low frequency and no rs5030737 variant has been found in the Chinese population, so it was excluded. The other three variants are localized in the promoter 1 (position–550, H/L variant (rs11003125) and –221, X/Y variant) and in the 5′ un-translated region (position +4, P/Q variant) of the *MBL2* gene. Rs11003125 is the main variant in the promoter region of the *MBL2* gene influencing serum *MBL* levels and is significantly associated with type 2 diabetes [2], [11]. Finally, three SNPs (rs1800450, rs1800451, and rs11003125) were selected. The three SNPs were determined in 1530 subjects by the Multiplex Snapshot method. The genotyping completion rate was 98% for the first experiment. The samples that were not successfully genotyped may have been polluted or uncertain factors existed, so we did the experiment again and the ultimate genotyping completion rate was 99%. Genotyping accuracy was demonstrated by showing a greater than 99.8% overall concordance rate in 10 blinded duplicate samples. The primers of the three SNPs were designed with Primer 5.0 software (Table S1) and synthetized by Invitrogen (Shanghai, China). Some biochemical indexes, such as fasting plasma-glucose, triglycerides and total cholesterol were also measured.

### Measurement of Serum *MBL* Levels

*MBL* concentrations were measured with enzyme-linked immune-sorbent assay (ELISA) (human *MBL* DuoSet, RD Systems). To guarantee the credibility and accuracy of the data of the assay, a spike and recovery immunoassay sample validation protocol was applied. High quality Bovine Serum Albumin (BSA, Sigma, USA) and 10% fetal calf serum (USA) were used as reagent diluents. The protocol showed that the spike recovery rate was in the range of 90%–120% and the sample exhibited good linear dilution, which indicated that no component in the samples interfered in ELISA. The serum concentrations of *MBL* were from the 675 patients.

### Statistical Methods

Allele and genotype frequency distributions for rs1800450 and rs11003125 were compared between controls and patients as well as patients grouped according to nephropathy status. Linkage disequilibrium and haplotype analyses were performed with Haploview 4.2. Odds ratio with a 95% confidential interval (*CI)* were calculated with SHesis software (http://analysis2.bio-x.cn/myAnalysis.php) [18]. Data were expressed as means and standard deviations (SD) for normally distributed variables, or medians and interquartile ranges (IQRs) for non-normally distributed variables. Between-group comparisons were performed with the *X^2^* test for categorical variables and the *T* test or Wilcoxon signed rank test for continuous variables. *P*<0.05 was considered to be statistically significant. All analyses were performed using SAS 9.1 (SAS institute Inc., Cary, NC).

## Results

Clinical characteristics of the study subjects are shown in Table 1. There was no gender difference between patients and controls (*P* = 0.13). Compared with controls, patients with type 2 diabetes had a significantly higher body mass index (BMI), waist-to-hip ratio (WHR), systolic blood pressure (SBP), triglyceride (TG), total cholesterol (TC), fasting plasma glucose (FPG), and creatinine (Cr) (*P*<0.001). Age, smoking status, and drinking status were also statistically significant between patients and controls. The clinical indexes were not significantly different among the three groups classified by nephropathy status except for gender, SBP, DBP, TC, Cr, and BUN.

**Table 1:** Basic characteristic of the study samples.

Variables	Type 2 diabetes	Controls	*P*	Type 2 diabetes			*P*
				Normal UAE	Microalbuminuria	Macroalbuminuria	
N	675	855		260	378	37	
Age(years)	62.0±12.7	64.3±10.3	<0.0001	62.5±11.9	62.1±12.8	57.3±15.2	0.06
Gender							
male	334	390		142	171	21	
female	341	465	0.13	118	207	16	0.04
Smoking status							
Yes	132	244		63	71	9	
No	543	611	<0.0001	197	307	28	0.23
Drinking status							
Yes	141	89		57	84	6	
No	534	766	<0.0001	203	294	31	0.70
BMI(kg/m^2^)	25.1±3.7	23.4±3.3	<0.0001	25.1±3.6	25.1±3.8	24.3±4.1	0.45
WHR	0.94±0.1	0.88±0.1	<0.0001	0.93±0.07	0.94±0.08	0.93±0.07	0.75
SBP(mmHg)	137.4±20.3	129.1±16.8	<0.0001	131.1±17.2	140.5±21.0	150.1±20.7	<0.0001
DBP(mmHg)	77.0±11.0	80.2±10.5	<0.0001	74.8±9.9	77.9±11.3	82.2±11.4	<0.0001
TG(mmol/L)	1.8±1.6	1.5±1.0	<0.0001	1.81±1.88	1.79±1.36	2.09±1.94	0.56
TC(mmol/L)	5.3±1.4	4.9±0.9	<0.0001	5.2±1.3	5.2±1.4	6.0±1.6	0.01
FPG(mmol/L)	8.5±3.5	5.2±0.8	<0.0001	8.6±3.1	8.6±3.8	7.9±3.4	0.48
Cr(µmol/L)	70(59–86)	68(54–83)	<0.0001	66(58–76)	73(58–93)	100(79–218)	<0.0001
BUN(mmol/L)	5.6(4.5–7.1)	5.9(4.6–10.9)	<0.0001	5.1(4.3–6.3)	5.7(4.5–8.0)	8.9(6.5–15.8)	<0.0001

BMI: body mass index; WHR: waist to hip ratio; SBP: systolic blood pressure; DBP: diastolic blood pressure; TG: triglyceride; TC: total cholesterol; FPG: fasting plasma glucose; Cr: creatinine; Bun: blood urea nitrogen; UAE: urinary albumin excretion in 24 h.

Data are shown as mean ± SD., median (interquartile range) or N.

### *MBL2* Gene with type 2 Diabetes and Diabetic Nephropathy

**Figure 1:**
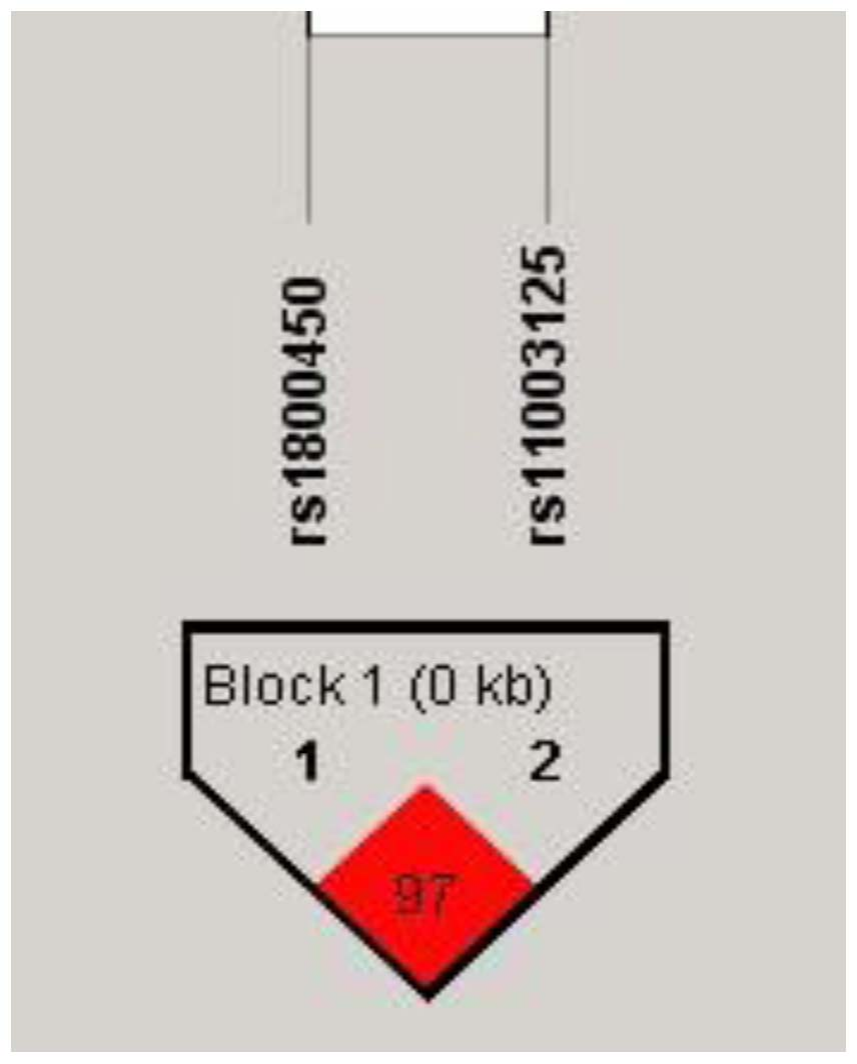
The LD displays plot of rs1800450 and rs11003125.

No mutation was found for rs1800451 in our study, which is consistent with previously published studies conducted in the Chinese population [16], [17]. The rs1800450 and rs11003125 were in Hardy-Weinberg equilibrium both in control subjects and patients with type 2 diabetes (Table 2). The rs1800450 and rs11003125 SNPs had strong linkage disequilibrium (r^2^ = 0.97) in our population and made up haplotype block 1 (Fig. 1).

**Table 2:** Hardy-Weinberger Equilibrium.

Polymorphisms	Genotype	Patients	*P*	Controls	*P*
Rs1800450	GG	502		588	
	GA	155		250	
	AA	18	0.155	17	0.104
Rs11003125	GC	350		411	
	GG	122		213	
	CC	203	0.175	231	0.264

The only haplotypes constructed from the risk-conferring (GC) allele showed increased trends for type 2 diabetes (*OR* = 1.2, 95% *CI*: 1.1–1.4, *P = *0.01, Table 3). For rs1800450 (Table 4), the GG genotype frequency was 74.4% in patients with type 2 diabetes vs. 68.8% in controls (*P* = 0.02), while the GA genotype frequency was 22.9% in patients with type 2 diabetes vs. 29.2% in controls (*P* = 0.01). For rs11003125, the GC genotype frequency was 18.07% in patients with type 2 diabetes vs. 24.91% in controls (*P = *0.01). The distributions of allele frequencies of rs1800450 and rs11003125 did not produce a statistical difference between patients and controls (*P* = 0.06, 0.84, respectively).

**Table 3:** Haplotype analyses of rs180045 and rs11003125.

Rs1800450	Rs11003125	Haplotype Frequency		*OR*(95% *CI*)	*P*
		Cases	Controls		
G	C	0.559	0.510	1.2(1.1–1.4)	0.01
G	G	0.300	0.324	0.9(0.8–1.0)	0.15
A	G	0.140	0.165	0.8(0.7–1.0)	0.06

**Table 4:** The distribution of genotype and allele frequency of *MBL2* in the study population.

Polymorphisms	Type 2 diabetes	Controls	*P*	Type 2 diabetes			*P*
				Normal UAE	Microalbuminuria	Macroalbuminuria	
N	675	855	–	260	378	37	–
Rs1800450							
GG	502(74.4)	588(68.8)	0.02	185(71.2)	289(76.4)	28(75.7)	0.32
GA	155(22.9)	250(29.2)	0.01	61(23.5)	64(22.8)*	8(21.6)	0.96
AA	18(2.7)	17(2.0)	0.38	14(5.3)	3(0.8)*	1(2.7)	0.01
G	1159(85.9)	1426(83.4)	–	431(82.9)	664(87.8)*	64(86.5)	–
A	191(14.1)	284(16.6)	0.06	89(17.1)	92(12.2)	10(13.5)	0.04
Rs11003125							
GG	350(51.9)	411(48.1)	0.14	52(20.0)	65(17.2)	5(13.5)	0.51
GC	122(18.1)	213(24.9)	0.01	131(50.4)	198(52.4)	21(56.8)	0.73
CC	203(30.0)	231(27.0)	0.19	77(29.6)	115(30.4)	11(29.7)	0.98
G	822(60.9)	1035(60.5)	–	235(45.2)	328(43.4)	31(41.9)	–
C	528(39.1)	675(39.5)	0.84	285(54.8)	428(56.6)	43(58.1)	0.76

* P<0.05 between patients with microalbuminuria and normal UAE.

The distributions of genotypes and allele frequencies among patients with normal UAE, microalbuminuria, and macroalbuminuria are shown in table 4. GA and AA genotype frequencies of rs1800450 were significantly different between patients with normal UAE and microalbuminuria, as well as for G and A alleles. No difference was found for rs11003125. Compared with other genotypes, subjects with the GG genotype of rs1800450 and the (GC+CC) genotype of rs11003125 had higher biochemical indexes, such as BMI, WHR, SBP, FPG, TG, TC and Cr, but no significant *p* value was found after adjustment for age, gender, smoking status and drinking status (Table S2).

### *MBL2* Gene and Serum *MBL* Levels

**Figure 2:**
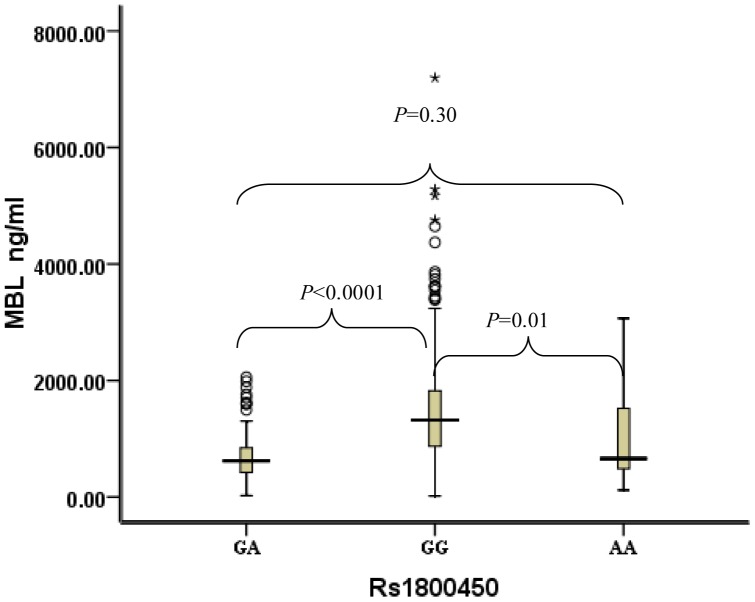
Distribution of serum MBL concentrations in type 2 diabetes stratified by rs1800450 genotypes. Black horizontal lines are median values; □ are interquartile ranges; ○ are outliers; * are extreme values. *P* values were results of pairwise comparison. The median MBL of the GA genotype was 621 ng/m (IQRs: 419–847), the median MBL of the GG genotype was 1322 ng/m (IQRs: 873–1823), and the median MBL of the AA genotype was 661 ng/m (IQRs: 488–1521).

**Figure 3:**
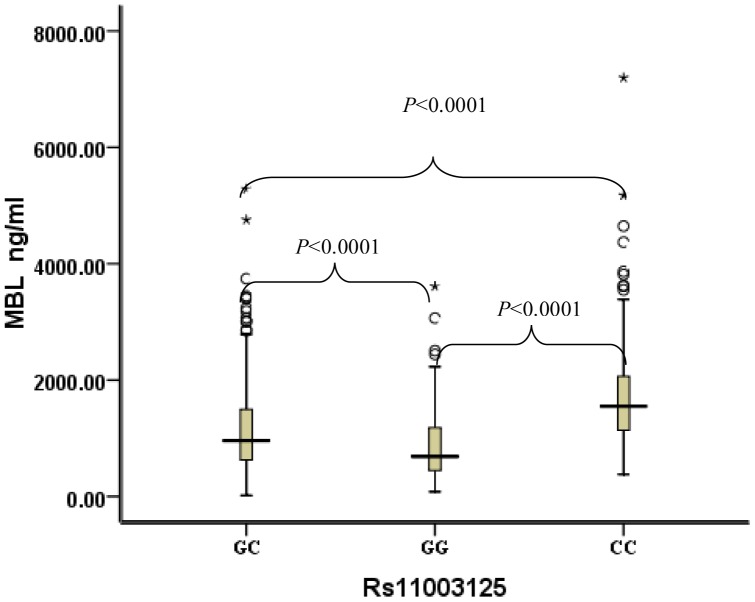
Distribution of serum MBL concentrations in type 2 diabetes stratified by rs11003125 genotypes. Black horizontal lines are median values; □ are interquartile ranges; ○ are outliers; * are extreme values. *P* values were results of pairwise comparison. The median MBL of the GC genotype was 962 ng/m (IQRs: 631–1498), the median MBL of the GG genotype was 690 ng/m (IQRs: 446–1181), and the median MBL of the AA genotype was 1553 ng/m (IQRs: 1130–2073).

For rs1800450 (Fig. 2), compared with subjects with GA and AA genotypes (median serum *MBL*: 621 ng/ml and 661 ng/ml), subjects with the GG genotype had much higher serum *MBL* levels (median: 1322 ng/ml). For rs11003125 (Fig. 3), compared with subjects with the GG genotype, subjects with the CC genotype had much higher serum *MBL* levels (median serum *MBL*: 1553 ng/ml vs. 690 ng/ml).

### Serum *MBL* Level and Diabetic Nephropathy

**Figure 4:**
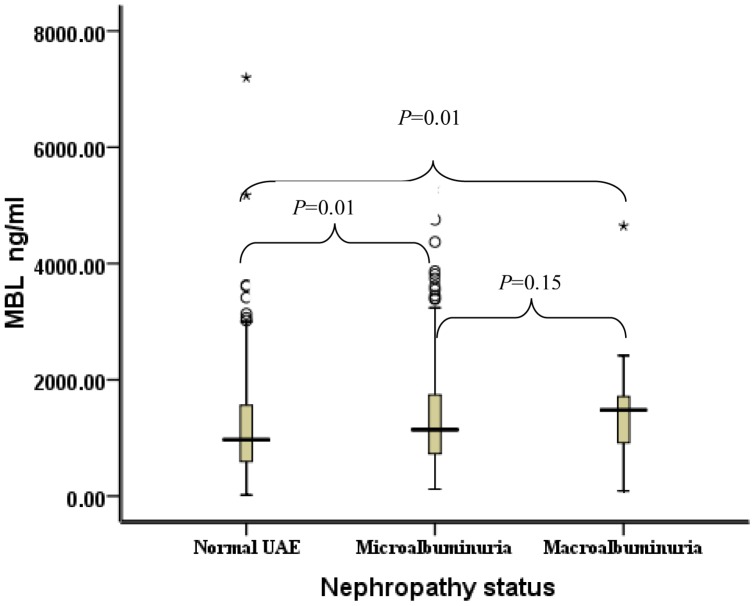
Distribution of serum MBL concentrations for patients stratified by nephropathy status. Black horizontal lines are median values; □ are interquartile ranges; ○ are outliers; * are extreme values. *P* values were results of pairwise comparison. The median MBL of patients with normal UAE was 971 ng/m (IQRs: 596–1560), the median MBL of patients with microalbuminuria was 1142 ng/m (IQRs: 728–1739), and the median MBL of patients with macroalbuminuria was 1482 ng/m (IQRs: 917–1713).

Compared with patients with normal UAE (median 971 ng/ml), patients with microalbuminuria and macroalbuminuria had much higher serum *MBL* levels (1142 ng/ml and 1482 ng/ml, respectively) and their differences were statistically significant (*P* = 0.01 for both). No significant *P* value was found between patients with microalbuminuria and macroalbuminuria (*P* = 0.15, Fig. 4).

## Discussion

### *MBL2* Gene and Type 2 Diabetes

Epidemiological studies have suggested that genetically determined variation in *MBL* levels influences the susceptibility and the course of different types of infectious, autoimmune, metabolic and cardiovascular diseases [19]–[22]. Most studies focused on the association of the *MBL2* gene with type 1 diabetes and the conclusions were controversial [2], [23], [24]. Few studies have discussed the association between the *MBL2* gene and type 2 diabetes. A report from the Diabetes Mellitus Insulin-Glucose Infusion in Acute Myocardial Infarction (DIGAMI 2) trial suggested that the distributions of *MBL* genotypes were similar to those known in the general population [25]. Because the results were blunted by traditional risk factors, the report did not resolve the issues of whether low or high serum *MBL* levels and which genotypes were harmful to patients with type 2 diabetes and myocardial infarction. Muller’s study showed that the association between genes and diabetes was restricted to the region in the *MBL2 gene*, which means that evidence of association with type 2 diabetes is more likely to be derived from the *MBL2* gene rather than from other genes. Their result demonstrated that in both Native Americans (number = 3723) and Old Order Amish (number = 486), the rs1800450 and rs11003125 SNPs (from nineteen tag SNPs of *MBL2* gene) contributed to type 2 diabetes susceptibility [11]. We also found that rs1800450 and rs11003125 were associated with type 2 diabetes. Compared with controls, the GG genotype and G allele of rs1800450 were more common in patients with type 2 diabetes, while the GA genotype and A allele frequencies were higher in controls. Haplotype analysis showed that subjects with the G allele of rs1800450 and C allele of rs11003125 had a 20% higher risk for type 2 diabetes. This evidence suggested that the GG genotype of rs1800450 may be harmful to patients with type 2 diabetes and the variation of rs1800450 (G→A) may be beneficial for individuals in reducing the risk of type 2 diabetes. Because rs11003125 is in high LD with other SNPs (within intro 2 and a flanking region of *MBL2* gene), the contribution of other functional SNPs cannot be ignored. Our study did not indicate whether the *MBL2* gene influences type 2 diabetes by affecting insulin secretion or insulin action. Additional studies are needed to investigate the impact of the *MBL2* gene on specific type 2 diabetes related pathways. The possible explanation of the contradictory results between the *MBL2* gene and diabetes may be that the variants of the *MBL2* gene have significant racial differences and only a few polymorphisms have been studied in relatively small samples.

### *MBL2* Gene and Diabetic Nephropathy

This study is the first study to examine the associations of the *MBL2* gene and type 2 diabetic nephropathy. In Kaunisto’s study, significant evidence of association was observed for rs920727 with patients with end-stage renal disease (ESRD) and normal UAE. However, after adjustment for the potential confounders no evidence of the association was observed [2]. In Hansen’s study, high *MBL* genotypes were significantly more frequent in diabetic patients with nephropathy than those with normal UAE, and the risk of having nephropathy given a high *MBL* genotype assessed by odds ratio (OR) was 1.52 [7]. In our study, GA and AA genotypes and allele frequencies of rs1800450 were statistically significant between patients with normal UAE and microalbuminuria, but the evidence was not strong enough to reach the conclusion that rs1800450 was associated with type 2 diabetic nephropathy. No evidence was found for rs11003125.

### MBL and Diabetic Nephropathy

In Hansen’s study, frequencies of the genotypes producing high serum *MBL* levels were more common in patients with diabetic nephropathy than patients with normoalbuminuria [7], which suggested that serum *MBL* levels may be involved in the pathogenesis of micro- and macrovascular complications. After following up 326 patients with type 2 diabetes for 15 years, Hansen found that measurement of serum *MBL* alone can provide prognostic information on mortality and the development of albuminuria [26]. A recent study has proved that elevated serum *MBL* in patients with type 2 diabetes indicates poor diabetic control and development of diabetic nephropathy, especially in combination with serum CRP [27]. The association between high serum *MBL* levels and diabetic nephropathy was also observed in the present study, which is in accordance with previous studies [2], [7], [8]. Type 1 and type 2 diabetes are different, but the association between serum *MBL* levels and diabetic nephropathy is consistent with previous studies suggesting that diabetic microvascular lesions may have a similar pathogenesis, regardless of type 1 diabetes or type 2 diabetes. Although the physiological mechanisms underlying the association of serum *MBL* levels with diabetic nephropathy are still unknown, it was previously shown that serum *MBL* plays a dual role in modifying inflammatory responses [11]. Deficiency of serum *MBL* has been linked to insulin resistance and obesity as a result of a chronic infectious state or low-grade inflammation [28]. Serum *MBL* levels also affect metabolic pathways through stimulating fatty acid oxidation in skeletal muscle, or reducing release of tumor necrosis factor- α, interleukin-1, and interleukin-6 [29], [30]. On the other hand, high levels of serum *MBL* could lead to an overly activated complement system inducing inflammation damage or interweaving a complex autoimmune process [19].

### *MBL2* Gene and *MBL* Concentrations

Although serum *MBL* levels may increase two- to threefold during acute phase response, the serum *MBL* levels remain genetically determined [9]. Both rs1800450 and rs11003125 of the *MBL2* gene showed strong associations with serum *MBL* levels in our study, which was consistent with previous studies [2], [7]. Subjects with the GG genotype of rs1800450 and the CC genotype of rs11003125 had much higher serum *MBL* levels than those with GA and AA genotypes. In addition, high levels of serum *MBL* were associated with diabetic nephropathy. This evidence proved the association of *MBL2* gene with type 2 diabetes from another aspect. Some variants may have an independent effect on serum *MBL* levels or tag other still unknown functional variants, so more SNPs of the *MBL2* gene are needed to better understand the association between the *MBL2* gene and serum *MBL* levels.

Our study had some limitations. First, we did not collect the medication history of oral hypoglycemic drugs in our questionnaire, which may influence the levels of serum *MBL*. Second, because only three SNPs of the *MBL2* gene were selected, the associations between the seven *MBL2* haplotypes and type 2 diabetes and diabetic nephropathy were not discussed.

In summary, rs1800450 and rs11003125 of the *MBL2* gene are associated with type 2 diabetes in the Chinese Han population, but not associated with diabetic nephropathy. The two SNPs have a strong impact on serum *MBL* levels, and serum *MBL* levels which may influence diabetic nephropathy.

## Supporting Information

Table S1 **The primer sequence of rs1800450, rs1800451 and rs11003125.**
(DOC)

Table S2 **Clinical phenotypes according to** ***MBL2*** **genotypes in 1,530 subjects under the dominant genetic model.**
(DOC)
