# Supplementary material for: Association of Levels of Mannose-Binding Lectin and the MBL2 Gene with Type 2 Diabetes and Diabetic Nephropathy
Source: PLoS One. 2013 Dec 20;8(12):e83059. doi: 10.1371/journal.pone.0083059 (PMC3869742; doi:10.1371/journal.pone.0083059)
Supplement: Table S1 — The primer sequence of rs1800450, rs1800451 and rs11003125. (DOC) [file pone.0083059.s001.doc]

Table S1 The primer sequence of rs1800450, rs1800451 and rs11003125

| SNPs | Primer | Primer sequence |
| --- | --- | --- |
| Rs1800450 | F^a^ | TGAGTATGGTGGCAGCGTCTTA |
|  | R^b^ | CGTACCTGGTTCCCCCTTTT |
|  | Y^c^ | TTTTTTTGCAAAGATGGGCGTGATG |
| Rs1800451 | F^a^ | TGAGTATGGTGGCAGCGTCTTA |
|  | R^b^ | CGTACCTGGTTCCCCCTTTT |
|  | Y^c^ | TACCTGGTTCCCCCTTTTCT |
| Rs11003125 | F^a^ | GCTGAAAGCTGGTGATCCAAA |
|  | R^b^ | GGAGGAGGATTCAAGGCAAGT |
|  | Y^c^ | TTTTTTTTTTTTTTTTTTTTTTTTTTTTTTTTTTTTTTTTT GCTTCCCCTTGGTGTTTTA |

^a^ forward primer; ^b^ reverse primer; ^c^ snapshot primer
