# Supplementary material for: Association of Levels of Mannose-Binding Lectin and the MBL2 Gene with Type 2 Diabetes and Diabetic Nephropathy
Source: PLoS One. 2013 Dec 20;8(12):e83059. doi: 10.1371/journal.pone.0083059 (PMC3869742; doi:10.1371/journal.pone.0083059)
Supplement: Table S2 — Clinical phenotypes according to MBL2 genotypes in 1,530 subjects under the dominant genetic model. (DOC) [file pone.0083059.s002.doc]

Table S2 Clinical phenotypes according to *MBL2* genotypes in 1,530 subjects under the dominant genetic model

| Variable | Rs1800450 | | *P^a^* | Rs11003125 | | *P^a^* |
| --- | --- | --- | --- | --- | --- | --- |
|  | GA+AA | GG |  | GC+CC | GG |  |
| N | 440 | 1090 | - | 1195 | 335 | - |
| BMI(kg/m^2^) | 23.9±3.6 | 24.2±3.5 | 0.37 | 24.2±3.5 | 23.9±3.6 | 0.58 |
| WHR | 0.90±0.1 | 0.91±0.1 | 0.01 | 0.91±0.1 | 0.90±0.1 | 0.42 |
| SBP(mmHg) | 130.7±18.2 | 133.6±19.0 | 0.33 | 133.3±19.0 | 130.8±18.3 | 0.12 |
| FPG(mmol/L) | 6.5±2.6 | 6.8±3.0 | 0.83 | 6.8±3.0 | 6.4±2.7 | 0.87 |
| TG(mmol/L) | 1.63±1.7 | 1.65±1.1 | 0.68 | 1.65±1.3 | 1.60±1.5 | 0.45 |
| TC(mmol/L) | 5.0±1.2 | 5.1±1.2 | 0.65 | 5.1±1.2 | 5.0±1.0 | 0.52 |
| Cr(μmol/L) | 5.6(4.6-8.3) | 5.8(4.6-8.1) | 0.32 | 5.8(4.6-8.3) | 5.5(4.4-7.8) | 0.29 |
| BUN(mmol/L) | 69(55-83) | 69(56-85) | 0.71 | 69(56-85) | 70(57-82) | 0.09 |

^a^ adjusted for age, gender, smoking status, and drinking status.
